# 4th Order LC-Based Sigma Delta Modulators

**DOI:** 10.3390/s22228915

**Published:** 2022-11-18

**Authors:** Evelyn Cristina de Oliveira Lima, Antonio Wallace Antunes Soares, Diomadson Rodrigues Belfort

**Affiliations:** 1 Graduate Program in Electrical and Computer Engineering, Federal University of Rio Grande do Norte, Natal 59078-970, RN, Brazil; 2 Department of Electrical Engineering, Federal University of Rio Grande do Norte, Natal 59078-970, RN, Brazil

**Keywords:** AD converter, Sigma Delta Modulator, LC filter

## Abstract

Due to the characteristic of narrow band conversion around a central radio frequency, the Sigma Delta Modulator (ΣΔM) based on LC resonators is a suitable option for use in Software-Defined Radio (SDR). However, some aspects of the topologies described in the state-of-the-art, such as noise and nonlinear sources, affect the performance of ΣΔM. This paper presents the design methodology of three high-order LC-Based single-block Sigma Delta Modulators. The method is based on the equivalence between continuous time and discrete time loop gain using a Finite Impulse Response Digital-to-Analog Converter (FIRDAC) through a numerical approach to defining the coefficients. The continuous bandpass LC ΣΔM simulations are performed at a center frequency of 432 MHz and a sampling frequency of 1.72 GHz. To the proposed modulators a maximum Signal-to-Noise Ratio (SNR) of 51.39 dB, 48.48 dB, and 46.50 dB in a 4 MHz bandwidth was achieved to respectively 4th Order Gm-LC ΣΔM, 4th Order Magnetically Coupled ΣΔM and 4th Order Capacitively Coupled ΣΔM.

## 1. Introduction

The Analog-to-Digital Converter (ADC) design is the big challenge of research in the field of Radio Frequency (RF) receivers for SDR and Cognitive Radio (CR) [1]. The main idea behind SDR is to replace several RF receivers with a unique multi-standard receiver, pushing most of the signal processing, such as down-conversion, filtering and channel selection to the digital domain [2].

**Figure 1:**
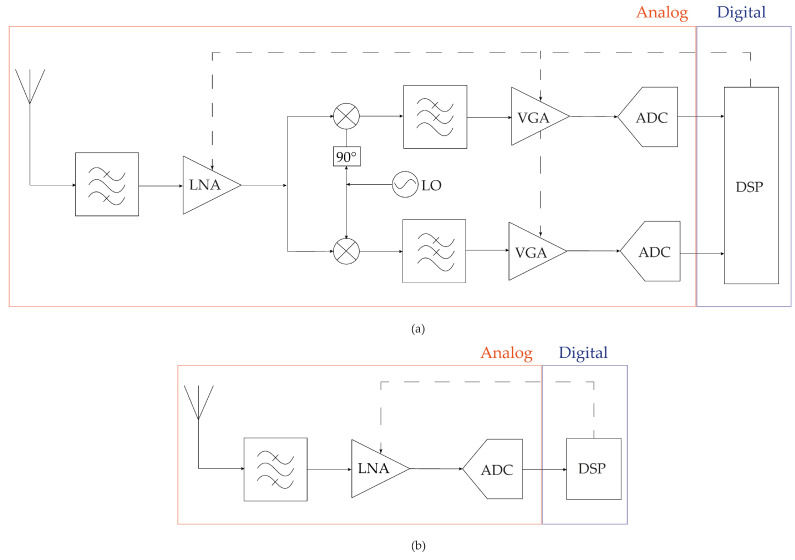
4th receiver architecture: (**a**) Conventional RF. (**b**) SDR based on RF ADC.

The specifications of the RF and analog circuits of the conventional RF receiver illustrated in Figure 1a, are tailored for a specific wireless communication standard. Thus, it is rather difficult to adapt the circuit’s specifications to different wireless standards. SDR receiver, illustrated in Figure 1b moves most of the functions performed by the tailored analog and RF circuits into the digital domain. In such an architecture the flexibility of the digital domain allows the radio to be easily configured, occasionally in real-time, to various standards, frequency bands, and bandwidths.

RF technology is used in many types of wireless devices, such as cell phones, radio and television broadcast stations, satellite communications systems, Wi-Fi, and Bluetooth. Table 1 summarizes the RF Applications in the 400–446 MHz band, which are explained as follows.

- The Industrial, Scientific and Medical(ISM) radio bands are reserved internationally to be used for industrial, scientific, and medical purposes other than communications [3]. In general, communications equipment operating in these bands must tolerate any interference generated by ISM equipment, and users have no regulatory protection from ISM device operation.
- Advanced Research and Global Observation Satellite (ARGOS) is a global satellite-based location and data-collection system dedicated to studying and protecting Earth’s environment [4]. It allows any mobile object equipped with a compatible transmitter to be located across the world.
- National Environmental Data System—“Sistema Nacional de Dados Ambientais” (SINDA) is the Brazilian data collection system, which has been built to collect environmental data such as temperature, pressure, and ultraviolet (UV) radiations from all Brazilian territory and provide this information to end users as hydroelectric power plants.
- The Medical Implant Communication Service (MICS) is used for diagnostic and therapeutic purposes in implanted medical devices in the human body. For example, MICS devices include implanted cardiac pacemakers and defibrillators as well as a neuromuscular stimulator that help restore sensation, mobility, and other functions to limbs and organs.
- Private Mobile Radio 446 (PMR446) is a part of the radio frequency range which is open and without licensing, used for business and personal use in most countries of the European Union.

The implementation of SDR using a Nyquist rate analog-to-digital converter (ADC) has been challenging and with very high-power consumption, which makes it unsuitable for mobile applications [5]. Bandpass ΣΔ modulators are well suited to SDR applications, since they are capable of converting a limited bandwidth centered on RF frequency. The center frequency $f_{o}$ is usually chosen to be $f_{s}/4$. This allows the multiplication to be performed with a simple exclusive-or gate, facilitating the design of the following down-conversion mixer and the decimation filter [6].

CT bandpass ΣΔM using LC resonators are well suited for SDR, as they can convert a narrowband around a Radio Frequency (RF) center frequency. In [7,8] RF LC-based 2nd order bandpass SD ADC has achieved interesting performances with Figure of Merits, but the obtained performances are not sufficient to be used in an RF transceiver for wireless communication standards. One way to increase the maximum achievable SNR of the SD ADC is by increasing the order of the loop filter of the SDM. In LC-based SDMs, increasing the order of the loop filter is usually done by cascading two or three LC resonators coupled using transconductance (Gm).

Another important context that we can cite regarding the use of SDM is in the paradigm of edge computing (EC), which faces many challenges concerning energy efficiency, data quality, reliability, data and device security, and computational performance level. EC is an emerging data processing paradigm that processes data over local computing and communication infrastructure, such as sensors and mobile sensor networks, and only, if necessary, prepares the data and establishes a communication link with a data center or other CE [9].

In [10], a 4th-order SDM, clocked at 3.6 GHz and centered at 900 MHz, was presented. The SDM ADC architecture was composed of 2 LC tank circuits with Q-enhancement, 3 transconductors, 1 single-bit comparator, and 3 single-bit NRZ feedback DAC. To tune and calibrate the Sigma Delta loop filter, a simple algorithm, suitable for integration, was presented.

High-order LC-based ΣΔM in the literature use the nodes between the LC tanks either for feedforward or feedback coefficients to obtain the desired Noise Transfer Function (NTF). These nodes are usually a source of additional noise, non-linearity, and power consumption to the overall performance of the ΣΔM. In [2] it is presented a systematic technique for designing SDM using single block high order filters, where only the filter input and the output node are accessible to the designer, making no use of intermediate nodes.

It is proposed in [11] the use of FIRDAC to increase the degrees of freedom by placing many delayed coefficients at the same node, which could ideally be applied to any n-order filter.

In this work, will be presented the projects of three 4th LC-Based single-block ΣΔM: 4th Order Gm-LC ΣΔM, 4th Order Magnetically Coupled ΣΔM and 4th Order Capacitively Coupled ΣΔM. The proposed methodology to design the modulators is based on the equivalence between the CT loop gain and the DT loop gain [11,12] and applying a numerical approach [13].

## 2. Materials and Methods

**Figure 2:**
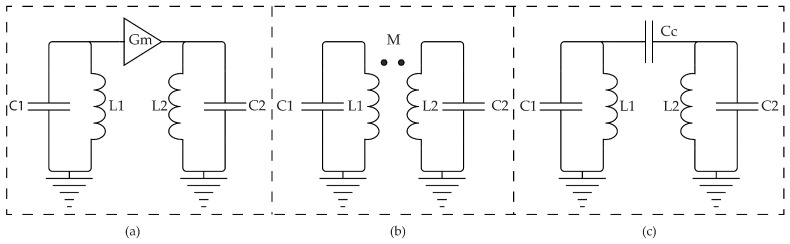
4th order LC filters using different coupling techniques: (**a**) Transconductance (Gm) coupling. (**b**) Magnetic coupling. (**c**) Capacitive coupling.

A high-order filter LC can be obtained using three main techniques of coupling that are shown in Figure 2.

The Gm coupling technique, for example, is based on converting the output voltage of the first tank into the input current of the second. This technique is generally applied to improve Gm linearity by the reason of it has an associated higher power consumption [14,15].

The Equation (1) defines a transfer function of two LC tanks that have the same inductors and the same capacitors where a transconductance Gm is placed between the two LC tanks, as shown in Figure 2a.

(1) $$H_{G_{m}-LC\left(s\right)}=\frac{G_{m}w_{0}^{4}L^{2}s^{2}}{{s^{2}+w_{0}^{2}}^{2}},$$

where,

(2) $$w_{0}=\frac{1}{\sqrt{LC}}.$$

**Figure 3:**
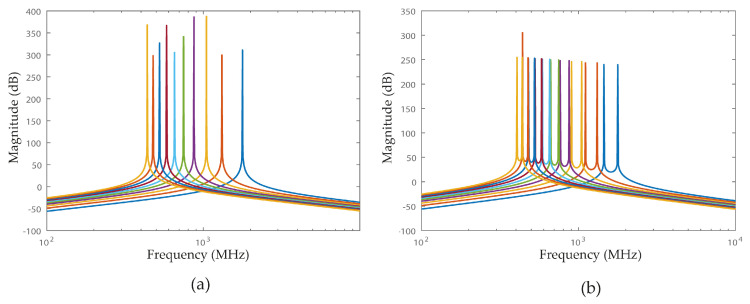
Frequency response of 4th order Gm coupled filters having: (**a**) $w_{1}=w_{2}=w_{0}$ (each line represents a center frequency ($w_{0}$) plotted for values of $C_{1}=C_{2}$ and $L_{1}=L_{2}$. With L values varying in the range 2 nH to 11 nH 2 and C values varying in the range 4 pF to 12 pF.). (**b**) $w_{1}\neq w_{2}$ (each line represents $w_{1}$ and $w_{2}$ to $L_{1}=L_{2}$ values varying in the range 2 nH to 11 nH, $C_{1}$ values varying in the range 4 pF to 12 pF, and $C_{2}$ values varying in the range 6 pF to 14 pF.).

Figure 3a shows the peak positioning for different center frequencies.

The Equation (3) is the transfer function that defines two LC tanks with the same inductor L and different capacitance. Where Gm is the transconductance placed between the two tanks and $w_{1}$ and $w_{2}$ are the resonance frequencies of the two LC.

(3) $$H_{G_{m}-LC\left(s\right)}=\frac{G_{m}w_{1}^{2}w_{2}^{2}L^{2}s^{2}}{\left(s^{2}+w_{1}^{2}\right)\left(s^{2}+w_{2}^{2}\right)}.$$

Figure 3b shows that the poles $w_{1}$ and $w_{2}$ are independent, and their position is directly defined.

Another technique of coupling is magnetic coupling, presented in Figure 2b. The working principle of this technique is the induction of the current in the second tank by a magnetic field due to current variation in the first one. From the occupied area point of view, this approach could be attractive, taking that a transformer can be easily implemented with two inductors occupying the same area on different metal layers. Some expertise concerning the used process technology is needed because the technical parameters are not always available and the translation to a specific tool is not a trivial task.

The transfer function of a magnetically coupled LC filter with identical LC tanks is defined by Equation (4):

(4) $$H_{MC}\left(s\right)=\frac{\frac{ksL}{\left(1-k^{2}\right)C^{2}L^{2}}}{s^{4}+s^{2}\frac{2}{(1-k^{2})CL}+\frac{1}{\left(1-k^{2}\right)C^{2}L^{2}}},$$

where k=M/L is the coupling factor and M is the mutual-inductance, $w_{0}$, the resonance frequency of the LC tanks and $w_{1}$ and $w_{2}$. The resonance frequencies of the 4th order magnetically coupled filter are defined like:

(5) $$w_{0}=\frac{1}{\sqrt{LC}},$$

(6) $$w_{1}=\frac{1}{\sqrt{(1+k)LC}},$$

(7) $$w_{2}=\frac{1}{\sqrt{(1+k)LC}}.$$

*k* in terms of resonance frequency, in terms of $w_{1}$ and $w_{2}$ is given by:

(8) $$k=\frac{\left({w_{2}}^{2}-{w_{1}}^{2}\right)}{\left({w_{1}}^{2}+{w_{2}}^{2}\right)}.$$

**Figure 4:**
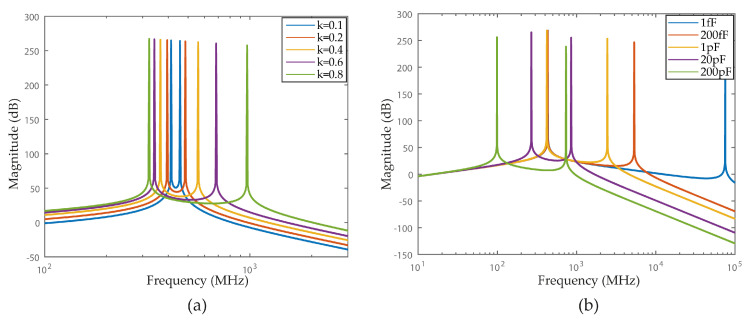
Frequency response of 4th order magnetically coupled LC filter: (**a**) With different values of the coupling factor k for $w_{0}=432$ MHz. (**b**) With a fixed k = 0.8, $C_{2}$ = 10.8 pF, L = 12.5 nH and different $C_{1}$.

It is important to note that the main issue of this topology, which is the high dependence on the coupling factor *k*: the resonance frequencies, $w_{1}$ and $w_{2}$, are a part from $w_{0}$ by a factor of 1/(1+k). Figure 4a shows poles spreading with respect to the coupling factor *k*, for $w_{0}$ = 432 MHz.

To achieve a narrowband (few MHz), the coupling factor should be very small (≈0.01). The coupling factor of integrated transformers normally varies between 0.5 and 0.8 [16]. It is possible to reduce the coupling factor by putting the coils apart [17], however, this solution has several disadvantages: (1) it is very hard to obtain very low coupling factors with a good precision [17]; (2) it is difficult to have reliable simulation results; (3) significant increase in the surface area of the filter.

A configuration of magnetically coupled LC filter is using non-identical LC tanks. In this case, we consider the same inductor value for both tanks, but two different capacitors $C_{1}$ and $C_{2}$, resulting in this transfer function:

(9) $$H_{MC}\left(s\right)=\frac{\frac{ksL}{\left(1-k^{2}\right)C_{1}C_{2}L^{2}}}{s^{4}+s^{2}\frac{C_{2}+C_{1}}{\left(1-k^{2}\right)C_{1}C_{2}L}+\frac{1}{\left(1-k^{2}\right)C_{1}C_{2}L^{2}}},$$

and $w_{01},w_{02},w_{1},w_{2}$, the resonance frequencies of the first and the second tanks and the poles of the 4th order magnetically coupled LC filter are respectively:

(10) $$w_{0_{1}}=\frac{1}{\sqrt{LC_{1}}},$$

(11) $$w_{0_{2}}=\frac{1}{\sqrt{LC_{2}}},$$

(12) $$w_{1}=\sqrt{2}\sqrt{\frac{1}{(C_{1}+C_{2}+\sqrt{{\left(C_{1}-C_{2}\right)}^{2}+4C_{1}C_{2}k^{2}})L}},$$

(13) $$w_{2}=\sqrt{2}\sqrt{\frac{1}{(C_{1}+C_{2}-\sqrt{{\left(C_{1}-C_{2}\right)}^{2}+4C_{1}C_{2}k^{2}})L}}.$$

Through the equations below, it is possible to define a “forbidden zone”, where the poles cannot be placed. This zone is located between $w_{1_{max}}$ and $w_{2_{min}}$:

(14) $$w_{1_{max}}=\left\{\frac{1}{\sqrt{LC_{2}}},\frac{1}{\sqrt{LC_{1}}}\right\},$$

(15) $$w_{2_{min}}=\left\{\frac{1}{\sqrt{1-k^{2}}\sqrt{LC_{2}}},\frac{1}{\sqrt{1-k^{2}}\sqrt{LC_{1}}}\right\}.$$

In Figure 4b, we plotted the frequency response of a 4th order magnetically coupled LC filter with a fixed $k,C_{2}$ and L and different $C_{1}$.

Capacitively coupled LC filters, as in Figure 2c, can be described by the transfer function in Equation (16) when identical resonators are used. Thus, we have that:

(16) $$H_{cc}\left(s\right)=\frac{s^{3}\frac{C_{c}}{C(C+2C_{c})}}{s^{4}+s^{2}\frac{2(C+C_{c})}{C(C+2C_{c})L}+\frac{1}{C\left(C+2C_{c}\right)L^{2}}}.$$

Assuming that C and L are respectively capacitance and inductance of the tank circuit, and $C_{c}$ is the coupling capacitance, from Equation (16), $w_{1}$ and $w_{2}$ can be defined as:

(17) $$w_{1}=\frac{1}{\sqrt{LC}},$$

(18) $$w_{2}=\frac{1}{\sqrt{\left(L\left(C+2C_{c}\right)\right)}}.$$

The $w_{1}$ and $w_{2}$ represent respectively the frequency of oscillation of the two tanks and the oscillation frequency due to the coupling capacitor. One can observe that, as $C_{c}$ tends to infinity, one pole is fixed, $w_{1}$, and the other pole tends to zero, $w_{2}$.

In the case of two different tanks, the transfer function used to describe the capacitively coupled LC resonators filter is:

(19) $$H_{cc}\left(s\right)=\frac{s^{3}\frac{C_{c}}{C_{1}C_{2}+C_{1}C_{2}c+C_{2}C_{c}}}{s^{4}+s^{2}\frac{C_{1}+C_{2}+2C_{c}}{(C_{1}C_{2}+C_{1}C_{2}c+C_{2}C_{c})L}+\frac{1}{\left(C_{1}C_{2}+C_{1}C_{2}c+C_{2}C_{c}\right)L^{2}}}$$

and the poles are positioned at:

(20) $$w_{1}=\sqrt{2}\sqrt{\frac{1}{C_{1}+C_{2}+2C_{c}-\sqrt{{\left(C_{1}-C_{2}\right)}^{2}+4{C_{c}}^{2}}}},$$

(21) $$w_{2}=\sqrt{2}\sqrt{\frac{1}{C_{1}+C_{2}+2C_{c}-\sqrt{{\left(C_{1}-C_{2}\right)}^{2}+4{C_{c}}^{2}}}}.$$

The minimum distance between the poles is achieved when the capacitance values are equal, being that minimum distance defined by the coupling capacitor. In general, a big coupling capacitor increases the minimum distance.

**Figure 5:**
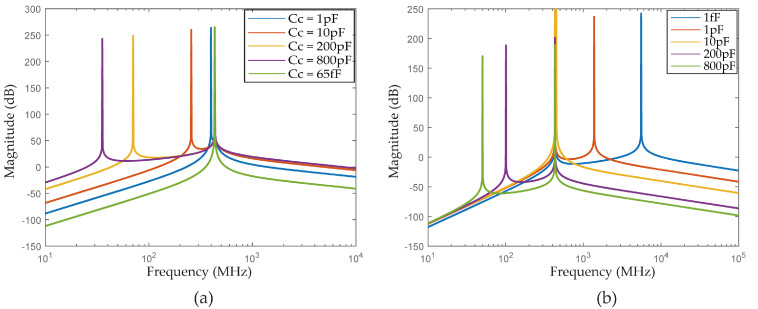
Poles positioning of 4th order capacitively coupled filter with: (**a**) Identically tanks. (**b**) Different tanks.

It is illustrated in Figure 5b the poles positioning of 4th order capacitively coupled filter with different tanks with a $C_{c}$ = 65 fF, L = 12.5 nH, $C_{2}$ = 10.8 pF and different values for $C_{1}$ (1 fF, 1pF, 10 pF, 200 pF, and 800 pF). One can see that the distance between the poles varies with the values of $C_{1}$ and that the minimum distance is reached when $C_{1}$ equals $C_{2}$.

### DT to CT Equivalence

**Figure 6:**
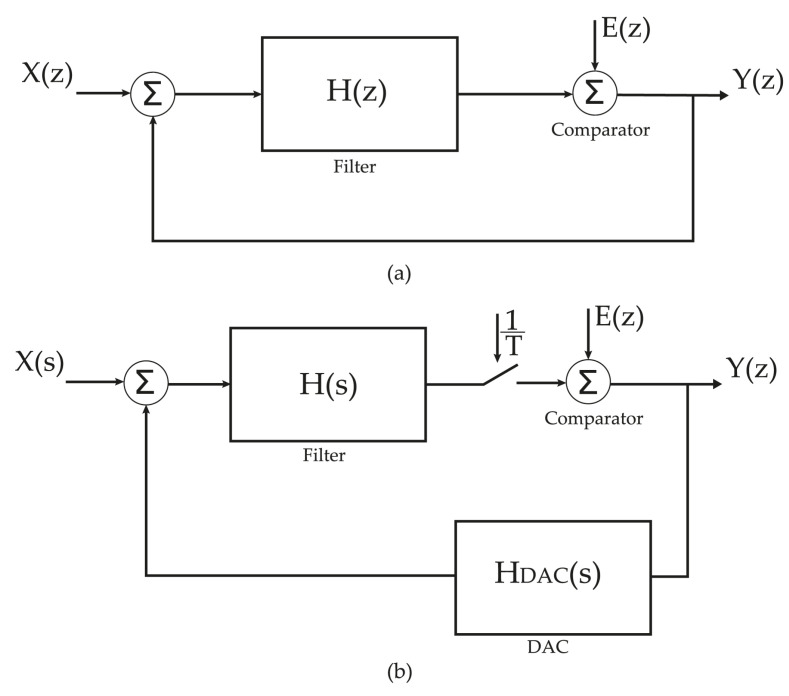
Equivalence between CT-DT ΣΔM: (**a**) Equivalent DT ΣΔM. (**b**) CT ΣΔM.

The 4th order LC filters presented above can be used as a loop filter of a 4th order bandpass ΣΔD Modulator. It is possible to design an ΣΔM with a 4th order CT based on the equivalence between the Continuous Time loop gain and the Discrete Time loop gain [18,19]. To this approach, the design of a CT ΣΔM start with the calculation of the DT equivalent model, as shown in Figure 6 [13]. That is, converting the loop gain from s-domain to z-domain through the impulse invariant transformation [20]:

(22) $$G_{C}\left(z\right)=Z\left\{L^{-1}{\left[H\left(s\right)H_{DAC}\right]}_{t=nT_{s}}\right\},$$

where H(s) is the transfer function of the loop filter, $H_{DAC}\left(s\right)$ is the feedback Digital-to-Analog Converter (DAC) transfer function and $T_{s}$ is the sampling time. The operators *Z* and $L^{-1}$ represent the *Z* transform and Inverse Laplace Transform, respectively.

The loop Gain of the DT ΣΔM can be designed through Schreier toolbox [21]. The “synthesizeNTF” function provides the NTF that allows us to calculate the DT loop gain:

(23) $$G_{D}\left(z\right)=\frac{1-NTF(z)}{NTF(z)}.$$

**Figure 7:**
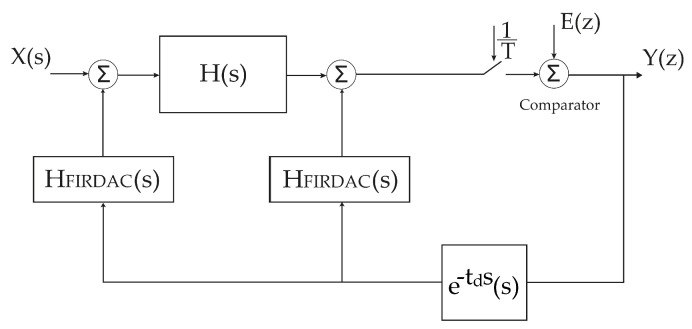
Continuous-Time SDM using FIRDACs.

The loop gain, $G_{C}\left(z\right)$, is not optimal and needs to be modified to match the optimal loop gain of the DT ΣΔM of the same type and order. A technique to match CT LG to DT LG trough FIRDACs (Equation (24)) was proposed in [11,12], where the feedback loop is composed of two FIRDACs, the first one in the main feedback path and the second one between the output of the comparator and its input to compensate the DAC delay, as illustrated in Figure 7. This approach is based on equating the partial fractions of both sides to calculate the FIR coefficients, so despite being accurate, it is very difficult to generalize, due to the complicated formulas used.

(24) $$G_{D}\left(z\right)=G_{C}\left(z\right)\cdot F\left(z\right).$$

In [13] a numerical approach is proposed to overcome the complexity of the analytical equations of the questions found in [11,12]. For this approach, the Equation (24) is reduced because, once the denominators are equal, the equation can be written only in terms of numerators:

(25) $$B_{D}\left(z\right)=B_{C}\left(z\right)\cdot F\left(z\right).$$

By expanding and writing the multiplication in matrix format, it results in:

(26) $$\left[\begin{array}{c} b_{D0}\\ b_{D1}\\ \vdots \\ b_{Dn}\\ 0\\ \vdots \\ 0 \end{array}\right]=\left[\begin{array}{ccccc} b_{C0} & 0 & 0 & \cdots & 0\\ b_{C1} & b_{C0} & 0 & \cdots & 0\\ b_{C2} & b_{C1} & b_{C0} & \cdots & 0\\ \vdots & \vdots & \vdots & \cdots & \vdots \\ b_{Ck} & b_{Ck-1} & b_{Ck-2} & \cdots & b_{C0}\\ 0 & b_{Ck} & b_{Ck-1} & \cdots & b_{C1}\\ \vdots & \vdots & \vdots & \cdots & \vdots \\ 0 & 0 & 0 & \cdots & b_{Ck} \end{array}\right]=\left[\begin{array}{c} f_{0}\\ f_{1}\\ \vdots \\ f_{m} \end{array}\right]$$

Finally, the FIR coefficients are calculated directly using matrix division:

(27) $$\left[f\right]={\left[b_{C}\right]}^{-1}\cdot \left[b_{D}\right].$$

## 3. Results

**Figure 8:**
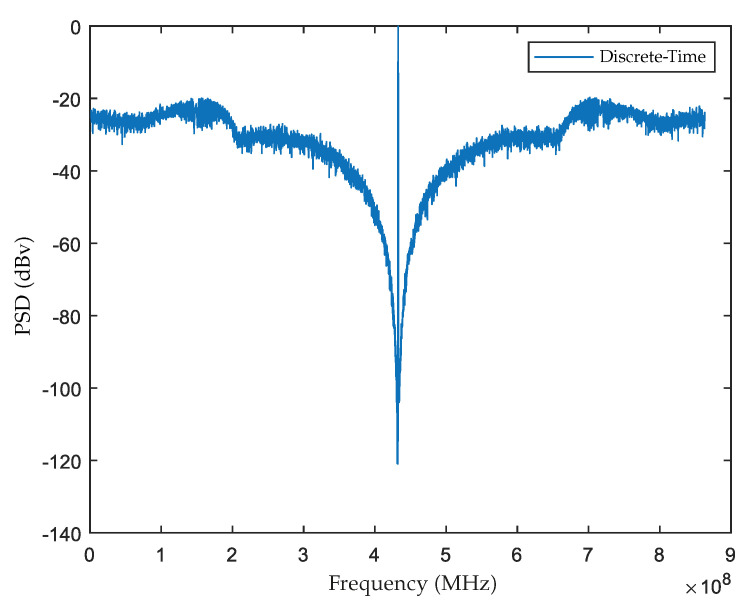
Output Spectrum of the designed DT ΣΔM.

The design of DT ΣΔM was done by using the “synthesizeNTF” function of Sigma Delta Toolbox on Matlab [21]. An Oversampling rate (OSR) of 64, a maximum gain of NTF of 1.5, and optimized NTF zeros were determined as parameters. For this DT design, the maximum SNR obtained was about 65 dB, the Power Spectral Density obtained is presented in Figure 8 and the DT Loop Gain is:

(28) $$G_{D}\left(z\right)=\frac{0.77^{-2}+0.56z^{-4}}{1+2z^{-2}+z^{-4}}.$$

The next step is to design the CT LC filter such that CT filter poles are coinciding with DT poles. In this work, it is defined that the coupling transconductance $G_{mc}$ between the two tank circuits is 10 nH, the center frequency at 432 MHz, and the poles placed at 430 MHz and 434 MHz using a 12.5 nH inductor. The transfer function $H_{DAC}$ of the rectangular NRZ feedback DAC is given by:

(29) $$H_{DAC}\left(s\right)=\frac{1}{s}\left(1-e^{-sT_{s}}\right).$$

### 3.1. 4th Order Gm-LC ΣΔM

The first CT LC filter is shown in Figure 1a and have like transfer function, the Equation (3).

Substituting from (3) and (29) into (22), the DT equivalent is calculated using Matlab^®^ function “c2d” with “impulse sampling” option:

(30) $$G_{C_{Gm}}\left(z\right)=\frac{0.9^{-2}-0.9z^{-3}-0.9z^{-4}+0.9z^{-5}}{1+2z^{-2}+z^{-4}}.$$

Substituting from (28) and (30) into (26) but, adding the coefficients of the compensation FIRDAC, we have:

(31) 00−0.770−0.55000000000=−0−0−0−00000−0−0−0−00000−0.9−0−0−01000−0.9−0.9−0−00100−0.9−0.9−0.9−02010−0.9−0.9−0.9−0.90201−0−0.9−0.9−0.91020−0−0−0.9−0.90102−0−0−0−0.90010−0−0−0−00001−0−0−0−00000−0−0−0−00000−0−0−0−00000−0−0−0−00000.fm0fm1fm2fm3fc0fc1fc2fc3

**Figure 9:**
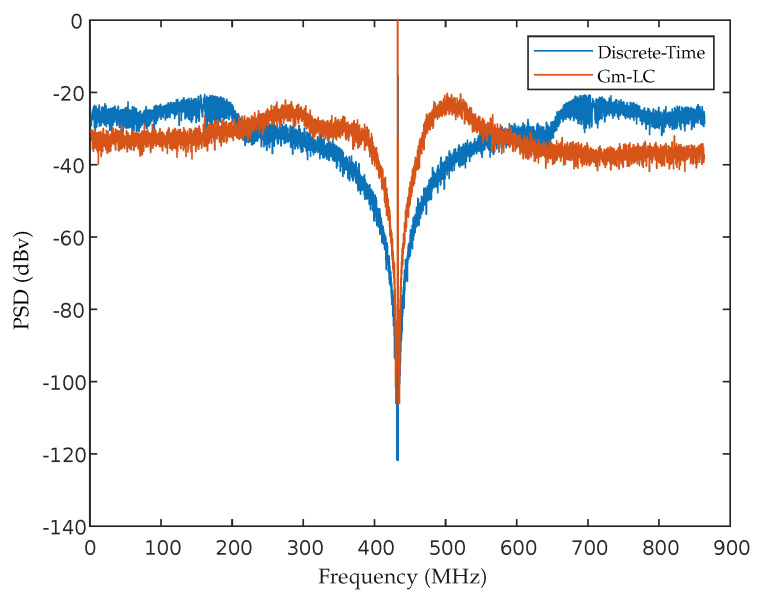
Output Spectrum of the designed Gm−LC ΣΔM.

So the coefficients can be computed. Table 2 shows the coefficients obtained for the 4th order Gm-LC coupled ΣΔM. Its PSD is depicted in Figure 9 and the maximum achieved SNR was about 51.39 dB.

### 3.2. 4th Order Magnetically Coupled ΣΔM

The second CT LC filter is a 4th order magnetically coupled filter as shown in Figure 2b that has the Equation 4 as transfer function. Substituting from (4) and (29) into (22), the DT equivalent is calculated using Matlab^®^ function “c2d” with “impulse sampling” option:

(32) $$G_{C_{MC}}\left(z\right)=\frac{0.14z^{-2}+0.29z^{-3}-0.29^{-4}+0.14z^{-5}}{1+2z^{-2}+z^{-4}}.$$

Substituting from (28) and (32) into (26) but, adding the coefficients of the compensation FIRDAC, we have:

(33) 00−0.770−0.55000000000=−0−0−0−00000−0−0−0−00000−0.14−0−0−01000−0.29−0.14−0−00100−0.29−0.29−0.14−02010−0.14−0.29−0.29−0.140201−0−0.14−0.29−0.291020−0−0−0.14−0.290102−0−0−0−0.140010−0−0−0−00001−0−0−0−00000−0−0−0−00000−0−0−0−00000−0−0−0−00000.fm0fm1fm2fm3fc0fc1fc2fc3

**Figure 10:**
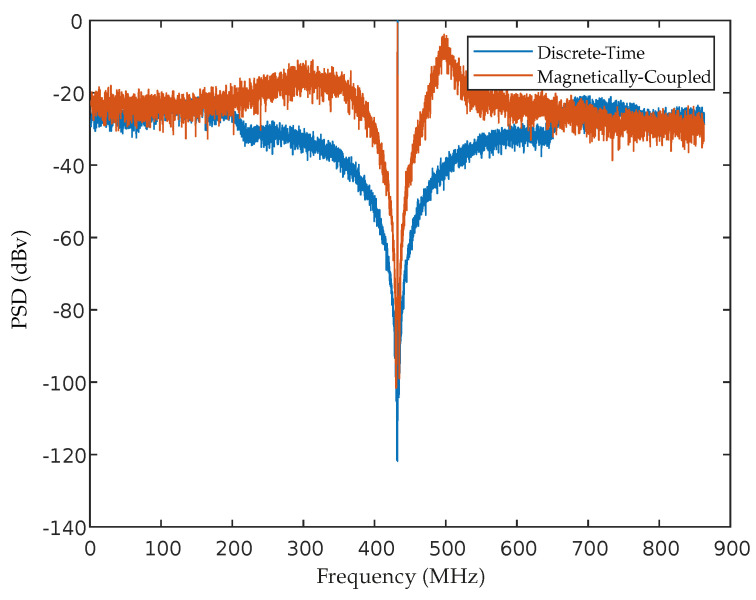
Output spectrum of the designed magnetically coupled ΣΔM.

The coefficients obtained for this design are shown in Table 3. The maximum achieved SNR was about 48.48 dB, and the PSD is depicted in Figure 10.

### 3.3. 4th Order Capacitively Coupled ΣΔM

The third CT LC filter is a 4th order capacitively coupled filter, as shown in Figure 2c. This filter has Equation (16) as a transfer function. Substituting from (16) and (29) into (22), the DT equivalent is calculated using Matlab^®^ function “c2d” with “impulse sampling” option:

(34) $$G_{C_{CC}}\left(z\right)=\frac{-0.16z^{-2}+0.65z^{-3}-0.65z^{-4}+0.16z^{-5}}{1+2z^{-2}+z^{-4}}.$$

Substituting from (28) and (34) into (26) and adding the coefficients of the compensation FIRDAC, we have:

(35) 00−0.770−0.55000000000=−0−0−0−00000−0−0−0−00000−0.16−0−0−01000−0.65−0.16−0−00100−0.65−0.65−0.16−02010−0.16−0.65−0.65−0.160201−0−0.16−0.65−0.651020−0−0−0.16−0.650102−0−0−0−0.161010−0−0−0−00001−0−0−0−00000−0−0−0−00000−0−0−0−00000−0−0−0−00000.fm0fm1fm2fm3fc0fc1fc2fc3

**Figure 11:**
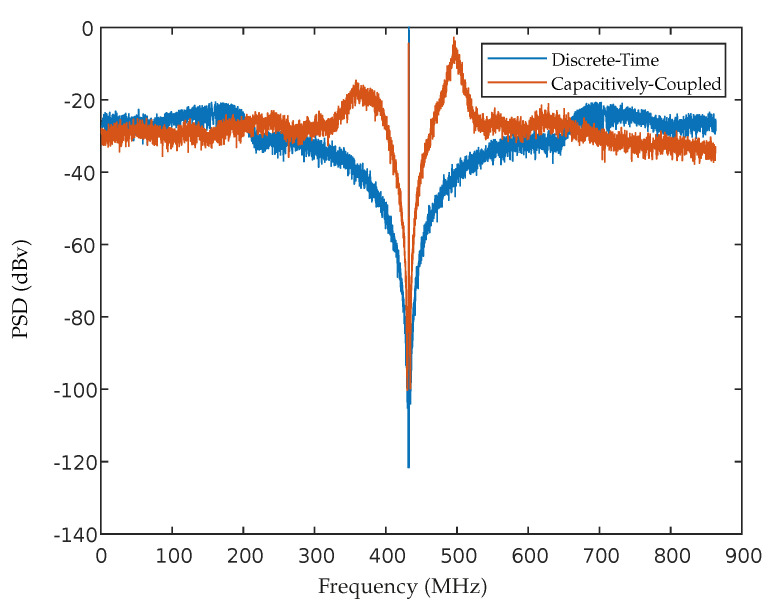
Output spectrum of the designed capacitively coupled ΣΔM.

The coefficients are computed and presented in Table 4, where is observed the coefficients obtained for the 4th order capacitively coupled ΣΔM. Its PSD is depicted in Figure 11 and the maximum achieved SNR was about 46.50 dB.

## 4. Discussion

Regarding the pole positioning in Gm-LC and the capacitive coupling architectures present, respectively, none and low dependency on the coupling device. In these architectures, the individual positioning of the poles can be easily done. On the other hand, poles positioning in magnetic coupling are extremely dependent on the coupling factor, creating a forbidden zone where the poles cannot be placed. This coupling depends on the distance between the two inductors and cannot be trimmed, which makes this architecture not robust to process variations if a low coupling coefficient value is needed.

Capacitive coupling uses a linear device, and magnetic coupling is modeled as one. The Gm-LC is naturally non-linear, due to the transistors that operate in saturation. Ideally, the power consumption for passive coupling architectures is zero, as well as their noise contribution.

**Figure 12:**
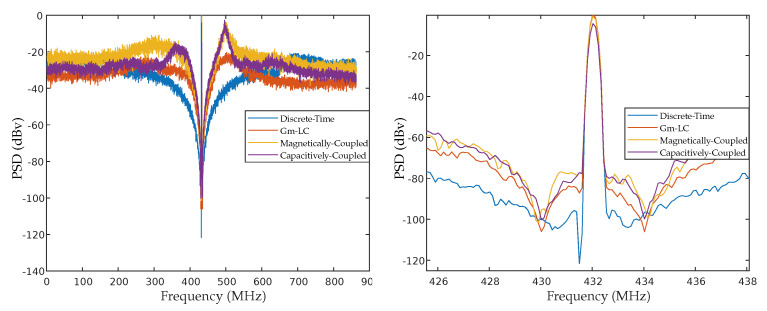
Output spectrum: (**a**) Of the all designed ΣΔMs. (**b**) Enlarged image around the center frequency.

Regarding 4th order ΣΔMs presented here, Figure 12a shows the comparison of the PSD for all three designed modulators and their equivalent DT for maximum SNR and Figure 12b the magnification of the image around the center frequency. All designed ΣΔMs had a good agreement with their DT equivalent as shown in, validating the proposed methodology.

Advances in highly integrated wireless communication transceivers provide applications for integrated RF bandpass filters. Active filters have a poor dynamic range when operating at high frequencies [22]. Passive LC filters can achieve high dynamic range at very low power consumption, but on-chip inductors have very low-quality factors, Q. Active LC filters are a good compromise between these two types of filters [23]. In active LC filters, the quality factor is enhanced by adding a negative resistance to compensate for the losses of the reactive devices. Active LC filters are not only used to realize integrated RF bandpass filters but they are also used in the design of RF bandpass SDMs. The methodology to design high-order LC-Based SDM introduced opens the door to unexplored filter topologies such as high-order passively coupled LC filters and Microelectromechanical Systems (MEMS) filters.

In Table 5, a comparison between the present work with other RF SDM implementations is presented. This table shows that the proposed architecture achieves good performances, to the state of the art.

The oversampling technology and noise shaping make Sigma Delta modulation have many potential applications, as demonstrated in [27], where an optical sigma-delta modulation theory is presented along with calculation and design issues for optical system implementation. It is demonstrated that Delta Sigma Modulation is a powerful technique for optical signal processing due to its high bandwidth, noise immunity, and cost. The Delta-Sigma modulation concept is also used by [28] in a system to resolve the influence of the reflecting surface of the deflection and the large-scale traction mobile feed system on the astronomical signal received by the feed-in FAST. Experimental results show that, when compared to traditional solutions, the quantization noise generated by the transmission end of the analog signal to the digital signal is reduced.

## 5. Conclusions

In this work, a design methodology for ΣΔM using a high-order realistic LC filter without local feedback was presented. This design methodology proposed the use of FIRDACs to increase the degrees of freedom, making possible the transformation of DT-to-CT. The desired loop-filter transfer function was designed taking into account, the LC resonators, the coupling coefficient, and the loop delay.

System-level simulation shows that the 4th order ΣΔMs using different coupling techniques achieves similar performances as their 4th order DT counterpart. For a real implementation, the difficulties of these architectures are related to the filter design, more specifically to the type of coupling used, and the capacitive coupling appears to be a good candidate for implementation. The capacitor is a well-known device, in addition, have good linearity, lower power consumption, and its design does not require other tools than a SPICE simulator.

## Figures and Tables

**Table 1:** Bands and standards in the 400–446 MHz range.

Ref.	Frequency Range (MHz)	Channel Bandwidth (kHz)	Application
ARGOS	401.635–401.665	30	Active RC
SINDA	401.605–401.635	30	Satellite
MICS	401.0–406.0	300	Medical
ISM-433	433.050–434.790	1840	any
PMR-466	446.0–446.2	12.5	Voice
KDR-444	444.6–444.975	16	Voice

**Table 2:** Coefficients for the 4th order Gm-LC coupled ΣΔM.

FIRDACs	Coefficient 0	Coefficient 1	Coefficient 2	Coefficient 3
FIRDACm	−0.27	−0.27	−0.21	−0.21
FIRDACc	−0.52	0.00	0.19	0.00

**Table 3:** Coefficients for the 4th order magnetically coupled ΣΔM.

FIRDACs	Coefficient 0	Coefficient 1	Coefficient 2	Coefficient 3
FIRDACm	−1.0	1.19	−0.85	0.94
FIRDACc	−0.61	0.15	0.13	0.00

**Table 4:** Coefficients for the 4th order capacitively coupled ΣΔM.

FIRDACs	Coefficient 0	Coefficient 1	Coefficient 2	Coefficient 3
FIRDACm	−0.82	1.19	−0.60	0.97
FIRDACc	−0.90	0.72	−0.15	0.00

**Table 5:** State of the art on RF SD ADC.

Ref.	[This Work]	[Ashry] [24] 2013	[Chae] [25] 2016	[Belfort] [2] 2017	[Sayed] [26] 2020
Architecture	Gm-LC | Magnetic Coupling | Capacitive Coupling	Gm-LC	Active RC	Capacitive Coupling	LC based
Order	4	4	6	4	2
Tuning range (GHz)	–	–	0.180–0.20	0.40–0.44	1.50–3.00
Sampling Frequency, $f_{s}$ (GHz)	1.72	0.36	0.80	0.58	6.00–12.00
Center Frequency, $f_{o}$ (GHz)	0.432	0.90	0.40–0.44	0.40–0.44	1.50–3.00
$f_{s}/f_{o}$	4	4	4–4/3	4/3	1/4
Bandwidth, BW (MHz)	4.00	28.13	25.55	4.50	47.00–93.00
SNR (dB)	51.39|48.48|46.50	50.00	69.00	50.00	37

## Data Availability

Not applicable.
